# Metabolomic biomarkers related to non-suicidal self-injury in patients with bipolar disorder

**DOI:** 10.1186/s12888-022-04079-8

**Published:** 2022-07-22

**Authors:** Xiangjie Guo, Jiao Jia, Zhiyong Zhang, Yuting Miao, Peng Wu, Yaqin Bai, Yan Ren

**Affiliations:** 1 Department of Forensic Medicine, Shanxi Medical University, Taiyuan, China; 2 Department of Psychiatry, Shanxi Bethune Hospital, Shanxi Academy of Medical Sciences, Tongji Shanxi Hospital, Third Hospital of Shanxi Medical University, 99 Longcheng street, Taiyuan, 030032 Shanxi China; 3 Tongji Hospital, Tongji Medical College, Huazhong University of Science & Technology, Wuhan, 430030 China; 4 Department of Psychology, School of Humanities and Social Sciences, Shanxi Medical University, Taiyuan, China; 5 Department of Health Statistics, School of Public Health, Shanxi Medical University, Taiyuan, China; 6 Shanxi Provincial Key Laboratory of Brain Science and Neuropsychiatric Diseases, Taiyuan, China

**Keywords:** Bipolar disorder, ^1^H-NMR technology, Metabolomics, Non-suicidal self-injury

## Abstract

**Background:**

Non-suicidal self-injury (NSSI) is an important symptom of bipolar disorder (BD) and other mental disorders and has attracted the attention of researchers lately. It is of great significance to study the characteristic markers of NSSI. Metabolomics is a relatively new field that can provide complementary insights into data obtained from genomic, transcriptomic, and proteomic analyses of psychiatric disorders. The aim of this study was to identify the metabolic pathways associated with BD with NSSI and assess important diagnostic and predictive indices of NSSI in BD.

**Method:**

Nuclear magnetic resonance spectrometry was performed to evaluate the serum metabolic profiles of patients with BD with NSSI (*n* = 31), patients with BD without NSSI (*n* = 46), and healthy controls (*n* = 10). Data were analyzed using an Orthogonal Partial Least Square Discriminant Analysis and a t-test. Differential metabolites were identified (VIP > 1 and *p* < 0.05), and further analyzed using Metabo Analyst 3.0 to identify associated metabolic pathways.

**Results:**

Eight metabolites in the serum and two important metabolic pathways, the urea and glutamate metabolism cycles, were found to distinguish patients with BD with NSSI from healthy controls. Eight metabolites in the serum, glycine and serine metabolism pathway, and the glucose-alanine cycle were found to distinguish patients with BD without NSSI from healthy controls. Five metabolites in the serum and the purine metabolism pathway were found to distinguish patients with BD with NSSI from those with BD without NSSI.

**Conclusions:**

Abnormalities in the urea cycle, glutamate metabolism, and purine metabolism played important roles in the pathogenesis of BD with NSSI.

## Background

BD represents a chronic and recurrent disorder that affects approximately 1% of the global population [1, 2]**,** resulting in a number of disabilities in young people, such as cognitive and functional impairment, in addition to increased mortality, particularly from suicide and cardiovascular disease [3, 4]**.** Bipolar disorder (BD) is characterized by biphasic moods that include depression and mania (in some cases, hypomania), which alternate throughout the course of the disease. Depressive episodes are more common in patients with BD, as a characteristic symptom, the incidence of NSSI is increasing gradually [5], which has important research significance.

Non-suicidal self-injury (NSSI), defined as the direct and deliberate destruction of one’s own bodily tissue (e.g., cutting, burning, and hitting oneself**)** in the absence of suicidal intent [6], has recently seen a sharp rise among young people [5]. As NSSI is the strongest predictor of future suicidal behavior [7, 8], it is important to probe its pathogenesis in patients with BD with NSSI. Following decades of progressive increase and growing scientific interest in the incidence of NSSI among adolescents and adults [5], NSSI is listed separately in the Diagnostic and Statistical Manual of Mental Disorders (fifth edition). However, there is little relevant research on the pathogenesis of NSSI. Studies suggested that the amygdala and nucleus accumbens may be potential treatment targets in persons who engage in NSSI [9, 10]. Meanwhile, Zahid, et al. have reported an association between neural activity across the dorsolateral prefrontal cortex and suicidal ideation and self-injury risk [11]. Related studies have also pointed to a relationship between abnormal gene methylation and NSSI [12, 13]. Studies have shown that increased inflammation may change major neurotransmitter metabolism, thereby affecting frontal function and decreasing response inhibition, which is associated with increased behavioral impulsivity. This may explain the neurobiological basis of NSSI. However, little is known about the neurobiological mechanisms and biomarkers of NSSI.

Metabolomics is a promising approach for the identification of potential diagnostic and treatment response biomarkers of psychiatric disorders [14, 15]. As blood samples are easier to obtain, it is possible to assess peripheral biomarkers. BD biomarkers in blood, serum, urine, and plasma have been probed using proton nuclear magnetic resonance (^1^H NMR) [16–18], gas chromatography-mass spectrometry [19], and in vivo brain imaging experiments [20, 21]. Significant metabolic markers, such as α-hydroxybutyrate, choline, and isobutyrate, among others, can differentiate patients with BD from healthy individuals [22–25]. However, little information on the biomarkers associated with NSSI is available. Therefore, a metabolomics study of blood samples may provide insightful information on the pathophysiology of BD. Thus, this study sought to assess the biomarkers of NSSI in patients with BD using metabolomics technologies.

## Methods

### Participants

This study was conducted at the Department of Psychiatry in Shanxi Bethune Hospital from January 2018 to August 2020. Inpatients and outpatients aged 15 to 45 years and diagnosed with BD during a depressive episode using the Diagnostic and Statistical Manual of Mental Disorders (fifth edition) were recruited. Participants were classified into two groups based on their history of NSSI: BD with NSSI (*n* = 31) and BD without NSSI (non-NSSI) (*n* = 46). Patients were directly interviewed, their medical records examined and medical information obtained. The Inventory of Statements About Self-injury (ISAS) assessment was conducted on all patients. Participants were asked if they had any self-injurious behaviors. Exclusion criteria: patients with suicidal ideation and behavior, cerebral trauma, other serious mental illness, and alcohol or substance abuse. The patients were diagnosed by at least two psychiatrists. The normal control group had no family history of psychiatric disorders. All participants were of Han Chinese origin.

### Collection of clinical data and assessment of patients

Demographic and clinical information of the participants was collected by a self-designed case data form, which include the following information: age, sex, onset age, family history, life events, sleep time, working pressure, and the presence or absence of NSSI and its specific situation.

The ISAS [26] comprehensively evaluates the characteristics and functions of NSSI. It consists of two parts. The first part evaluates the frequency of NSSI behavior, age at onset, pain experience, and resistance to NSSI behavior. Individuals with NSSI behavior were assessed in part II, including 13 potential functions: affect-regulation, anti-dissociation, anti-suicide, autonomy, interpersonal boundaries, interpersonal influence, marking distress, peer-bonding, self-care, self-punishment, revenge, sensation seeking, and toughness. The ISAS has high reliability and validity in measuring the frequency and function of NSSI. Patients who attempted NSSI more than 5 times in one year were classified into the NSSI group.

The HAMD-24 was used to measure the level of depression and changes in its severity. Patients with bipolar disorder was in a depressed state, as HAMD-24 score was > 17. The statistical results of related information are shown in Table 1.

**Table 1:** Demographic and clinical characteristics of all participants

Variable		Group			Analysis	
		BD (NSSI) (*N* = 31)	BD (Non-NSSI) (*N* = 46)	HC (*n* = 10)	F/χ^2^	p
Age (year)		25.48 ± 7.78	26.78 ± 9.94	26.9 ± 8.14	0.139	0.870
Onset age (year)		19.52 ± 5.56	22.09 ± 5.37	-	0.153	0.046*
The total scores of HAMD-24		37.32 ± 5.63	32.89 ± 5.36	-	0.040	0.001*
Number(NSSI)		6.06 ± 8.28	-	-		
Gender	male	11	21	3	1.288	0.525
	female	20	25	7		
marriage	1 = unmarried	28	26	7	11.185 (0.083)	
	2 = married	2	16	3		
	3 = divorced	1	3	0		
	4 = digamous	0	1	0		
	5 = cohabit	0	0	0		
	6 = widowed	0	0	0		
	7 = separated	0	0	0		
family history	1 = Yes	8	13	0	6.943	0.139
	2 = No	22	33	9		
	9 = unknown	1	0	1		
life events	1 = No	15	30	10	8.830	0.012*
	2 = Yes	16	16	0		
Sleep time	< 5 h	10	7	1	19.732	0.000*
	5-8 h	13	31	1		
	> 8 h	8	8	8		
working pressure	1 = No	10	27	7	10.877	0.028*
	2 = Mild	8	11	3		
	3 = severe	13	8	0		
way (NSSI)	1 = cutting	17	-	-		
	2 = scratching	8	-	-		
	3 = hitting	3	-	-		
	4 = biting	3	-	-		
Medicatio-n history	N	4	4			
	1 = Sertraline + lithium carbonate	2	1			
	2 = fluoxetine + lithium carbonate	1	1			
	3 = lithium carbonate	1	2			

### Collection of NMR metabolomics data

#### Serum collection

Participants were not allowed to eat or drink after 10 pm, then, serum samples were collected between 8:00 am and 9:00 am the next day.

#### ^1^H-NMR spectrum and data analysis

We used Bruker 600 MHz AVANCE III NMR spectrometer and Carr-Purcell-Meiboom-Gill (CPMG) pulse sequence. Parameter Settings are as follows: free induction attenuation (64 K data points), self-axonal relaxation delay (320 ms), 64 scans. In order to determine the related pathways of biomarkers, biomarkers will further import MetaboAnalyst 5.0 [27] (http://www.metaboanalyst.ca/), then, the pathway was screened by p-values of pathway enrichment analysis and Impact values of pathway topology analysis.

### Data processing and statistical analyses

SIMCA-P14.0 software was used to centralize and normalize the integral data collected and processed by NMR. Partial least squares discriminant analysis (PLS-DA) and orthogonal partial least squares discriminant analysis (OPLS-DA) were performed. Data are presented as mean ± standard deviation (SD). Statistical analyses comparing the two groups were performed using a two-tailed unpaired t-test, whereas two or more groups were compared using one-way ANOVA. *P* < 0.05 indicated a statistically significant difference.

## Results

### Demographic data of the participants

There were no significant differences in age (F = 0.139, *p* = 0.870), sex (χ2 = 1.288, *p* = 0.525), marital status (χ2 = 11.185, *p* = 0.083), or family history (χ2 = 6.943, *p* = 0.139) among the BD (NSSI), BD (non-NSSI), and HC (healthy control) groups. The BD (NSSI) group had a younger age at onset and higher total HAMD-24 scores than the BD (non-NSSI) group (F = 0.153, *p* = 0.046; and F = 0.040, *p* = 0.001, respectively). There were significant differences in life events (χ2 = 8.830, *p* = 0.012), sleep time (χ2 = 19.389, *p* = 0.001), and work pressure (χ2 = 10.877, *p* = 0.028) between the BD (NSSI) and BD (non-NSSI) groups (Table 1).

In the BD (NSSI) group, 61.3% of patients had frequent NSSI. The most common method of inflicting NSSI was cutting (54.8%), scratching (25.8%), hitting (9.7%), and biting (9.7%) (Table 1). Eight patients had previously used antidepressants and antipsychotic drugs––4 cases in NSSI group and 4 cases in non-NSSI group (Table 1); none of the other patients had taken any psychotropic drugs.

### Analysis of ^1^H NMR spectroscopy data

**Fig. 1:**
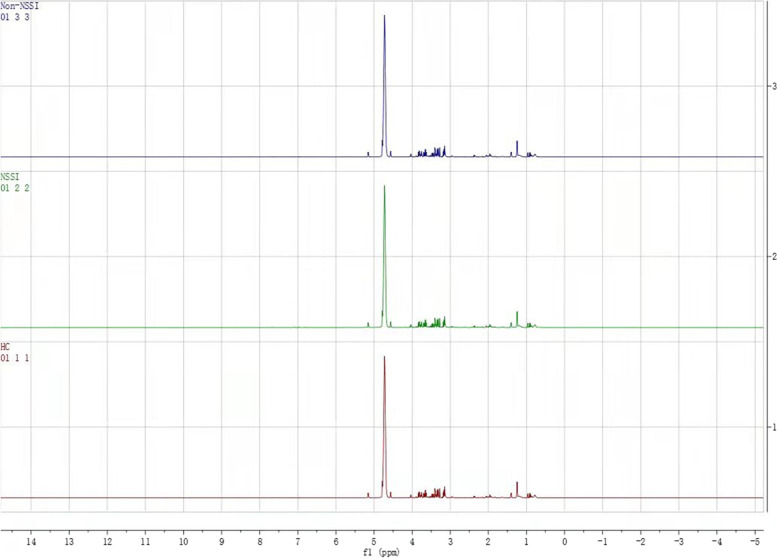
Proton nuclear magnetic resonance spectra of blood samples from patients with bipolar disorder and healthy controls

Figure 1 shows the typical ^1^H NMR spectrum profiles of the BD and HC groups. Twenty-eight small-molecule compounds were identified on the ^1^H NMR spectrum profiles of participants in the two groups based on the findings of the assessment of the human metabolome database (http://www.hmdb.ca/) and related articles published previously (Table 2).

**Table 2:** Peak attribution in 1H-NMR spectra of differential metabolites among three group

	Metabolites	Chemical shift
1	HDL	0.874(m)
2	pantothenate	0.907(s)
3	Isoleucine	0.949(t)
4	Leucine	0.961(t)
5	3-Hydroxybutyric acid	1.21(d)
6	Lactate	1.33(d)
7	Acetic acid	1.927(s)
8	O-Acetyl glycoproteins	2.14(s)
9	Acetoacetate	2.28 (s), 3.44 (s)
10	Methionine	2.14(s)
11	Guanidinoacetate	3.80 (s)
12	Uracil	5.81 (d, 7.7 Hz), 7.55 (d, 7.7 Hz)
13	Histidine	7.04 (s), 7.84 (s)
14	Dimethylglycine	2.92 (s), 3.70 (s)
15	Creatine	3.04 (s), 3.93 (s)
16	Acetylcholine	3.23 (s)
17	Taurine	3.27 (t, J = 6.6 Hz), 3.42 (t, J = 6.6 Hz)
18	Scyllo-inositol	3.36 (s)
19	3-D-hydroxybutyrate	1.20(d)
20	Betaine	3.27(m)
21	Betaine	3.67(m),3.78(m)
22	Citrulline	3.73(s)
23	N-Acetyl glycoproteins	2.05 (s)
24	Glutamate	2.06 (m), 2.14 (m), 2.36 (m)
25	Glutamine	2.14 (m)
26	Acetone	2.23 (s)
27	Citric acid	2.53 (d, 16.1 Hz), 2.70 (d, 16.1 Hz)
28	Choline	3.20 (s), 4.06 (m)

### Discriminative model construction

**Fig. 2:**
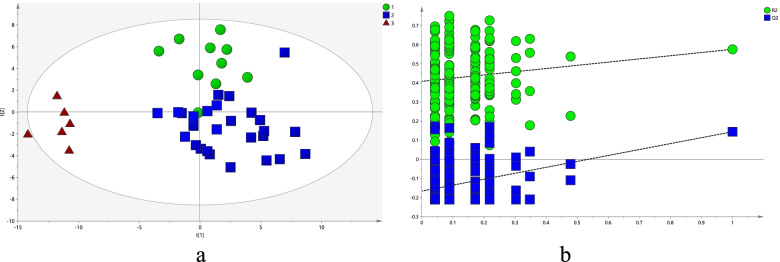
Proton nuclear magnetic resonance spectra of the partial least-squares discriminant analysis of the serum of patients in the three groups (circle: bipolar disorder with non-suicidal self-injury (NSSI) group; square: bipolar disorder without NSSI; triangle: healthy control group)

Partial least-squares discriminant analysis (PLS-DA) was used for ^1^H NMR metabolic profile analysis of all serum samples. The results were shown in Fig. 2a, indicating that the healthy control group was significantly separated from BD (NSSI) group and the BD (non-NSSI) group. Model verification results are shown in Fig. 2b. The results show that the PLS-DA model is effective.

### Differences in the plasma metabolite and metabolic pathways associated with the BD (NSSI) and HC groups

**Fig. 3:**
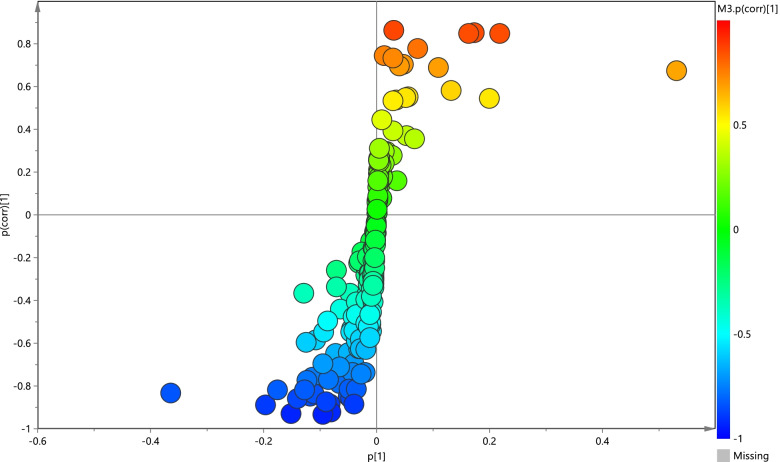
PCA plots of the OPLS-DA of the healthy control group and BD (NSSI) group, which were validated using S-plots

**Fig. 4:**
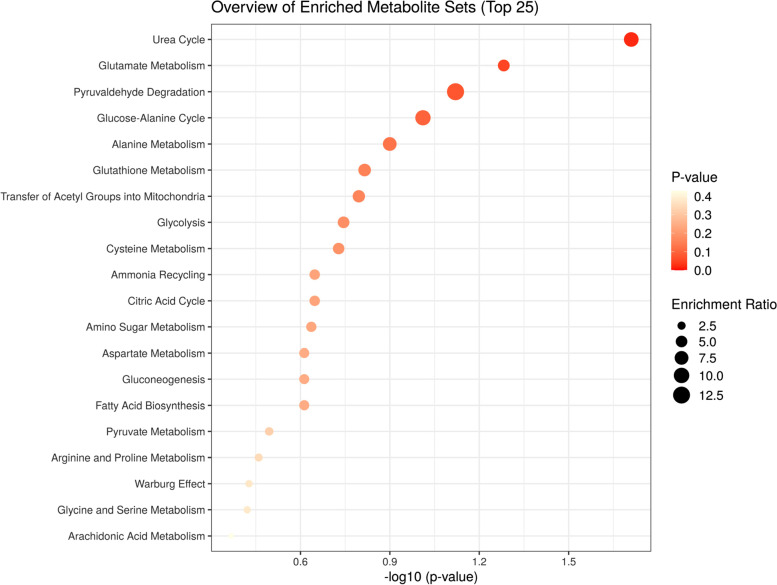
Metabolic pathways associated with bipolar disorder with non-suicidal self-injury

The corresponding OPLS-DA loading plot was further established between BD (NSSI) and HC groups (Fig. 3). A total of eight endogenous differential metabolites were detected (variable importance in projection [VIP] > 1, *P* < 0.05)—high-density lipoprotein (HDL), 3-hydroxybutyric acid, pyruvic acid, citrulline, and creatinine were significantly increased (*P < 0.05, **P < 0.01), while oxidized glutathione, glyceryl, and β-glucose were significantly decreased (*P < 0.05, **P < 0.01) in the BD (NSSI) group compared with that in the HC group (Table 3). Multiple differential metabolic pathways were identified, using an impact value above 0.05 (Fig. 4), including the urea cycle, the glutamate metabolism pathway, and the pyruvaldehyde degradation pathway.

**Table 3:** Peak area of metabolites in serum.^1^H-NMR spectra of HC and BD (NSSI) groups

Metabolites	Peak area after normalization	
	HC	BD (NSSI)
HDL	0.763 ± 0.102	1.170 ± 0.153^**^
3-Hydroxybutyric acid	0.477 ± 0.172	4.489 ± 1.854^**^
pyruvic acid	0.181 ± 0.025	0.556 ± 0.160^**^
oxidized glutathione	3.109 ± 0.434	1.694 ± 0.489^**^
Glyceryl	0.880 ± 0.087	0.711 ± 0.054^**^
Citrulline	0.746 ± 0.077	1.257 ± 0.219^*^
Creatinine	0.591 ± 0.116	1.213 ± 0.612^**^
β-glucose	40.352 ± 4.027	29.679 ± 6.850^**^

Relative peak area of potential biomarkers found in serum

Compared with control group: *P < 0.05, **P < 0.01

### Differences in the plasma metabolite and metabolic pathways associated with the BD (non-NSSI) and HC groups

**Fig. 5:**
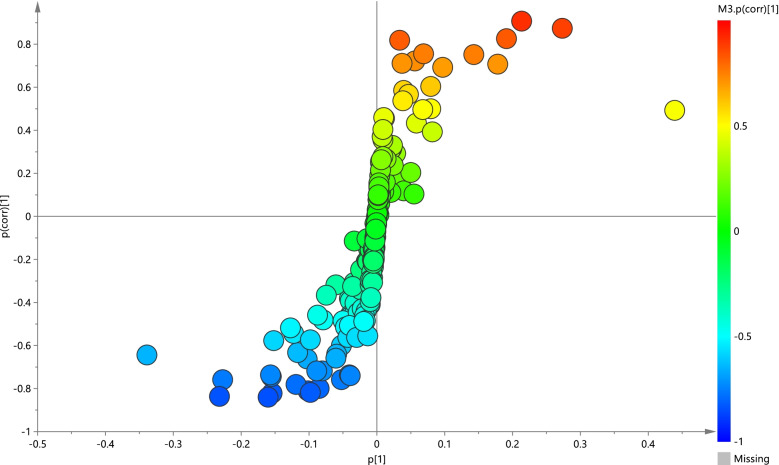
PCA plots of the OPLS-DA of the healthy control group and BD (non-NSSI) group, which were validated using S-plots

**Fig. 6:**
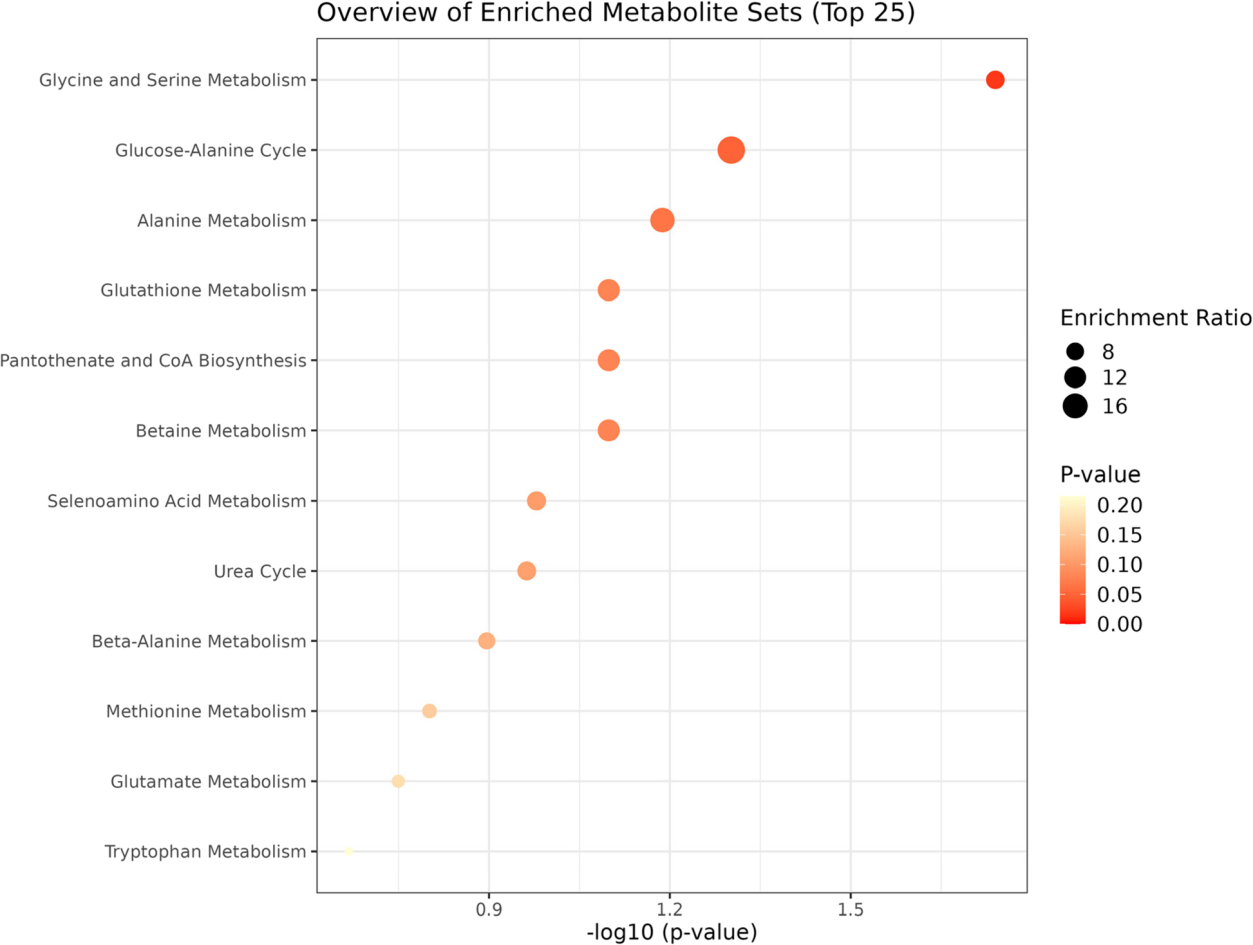
Metabolic pathways associated with bipolar disorder without non-suicidal self-injury

The OPLS-DA loading plot between BD (non-NSSI) and HC groups (Fig. 5) detected differences in a total of eight endogenous metabolites (VIP > 1 and *P* < 0.05)—HDL, pantothenate, alanine, glyceryl, dimethylglycine, and valine were significantly increased (*P < 0.05, **P < 0.01), and N-acetyl glycoproteins and ascorbate were significantly decreased (*P < 0.05, **P < 0.01) in the BD (non-NSSI) group compared with that in the HC group (Table 4). Through analysis of metabolic pathways, we found that the main metabolic pathways consisted of the glycine and serine metabolism pathway and the glucose-alanine cycle (Fig. 6).

### Differences between the plasma metabolites and metabolic pathways in the BD (non-NSSI) and BD (NSSI) groups

**Fig. 7:**
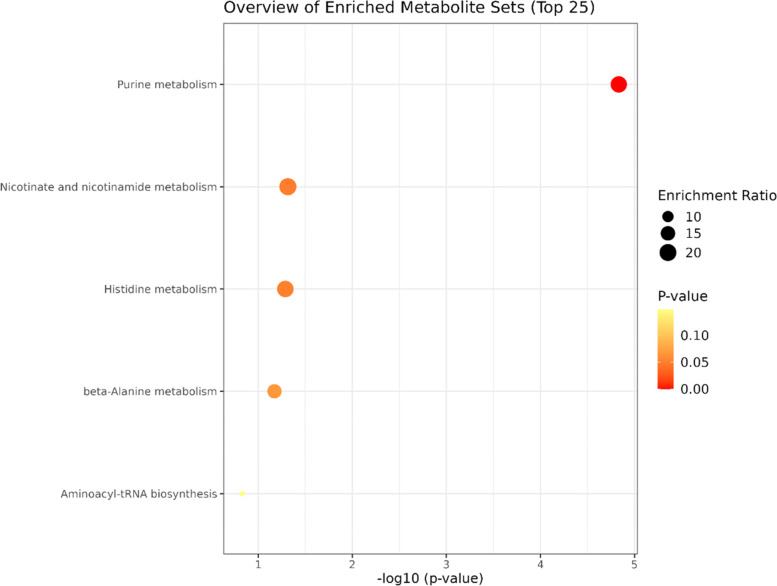
Comparison of metabolic pathways associated with bipolar disorder with and without non-suicidal self-injury

A total of five endogenous differential metabolites between the BD (non-NSSI) and the BD (NSSI) groups was identified (VIP > 1, *P* < 0.05)—xanthine, niacinamide, adenosine, hypoxanthine, and histidine were significantly higher in the BD (NSSI) group (*P* < 0.05, *P* < 0.01) (Table 5). Analysis of metabolic pathways revealed that the main differential metabolic pathways was the purine metabolism (Fig. 7).

**Table 4:** Peak area of metabolites in the serum.^1^H-NMR spectra of the HC and BD (non-NSSI) groups

Metabolites	Peak area after normalization	
	HC	BD (Non-NSSI)
HDL	0.699 ± 0.147	0.994 ± 0.149^**^
Pantothenate	0.472 ± 0.099	1.001 ± 0.165^**^
Alanine	0.058 ± 0.039	0.318 ± 0.218^**^
N-Acetyl glycoproteins	0.874 ± 0.162	0.641 ± 0.110^**^
Glyceryl	0.490 ± 0.078	1.956 ± 0.196^**^
Dimethylglycine	0.706 ± 0.123	1.349 ± 0.259^**^
Ascorbate	1.496 ± 0.263	0.680 ± 0.248^**^
Valine	0.035 ± 0.016	0.266 ± 0.082^**^

Relative peak area of potential biomarkers found in serum

Compared with control group: *P < 0.05, **P < 0.01

**Table 5:** Peak area of metabolites in the serum ^1^H-NMR spectra of the BD (NSSI) and BD (non-NSSI) groups

Metabolites	Peak area after normalization	
	BD(NSSI)	BD(Non-NSSI)
Xanthine	0.122 ± 0.322*	-0.008 ± 0.061
Niacinamide	0.040 ± 0.110*	-0.005 ± 0.036
Adenosine	0.103 ± 0.266*	0.001 ± 0.016
Hypoxanthine	0.120 ± 0.307*	0.002 ± 0.017
Histidine	0.094 ± 0.261*	-0.006 ± 0.050

Relative peak area of potential biomarkers found in serum

Compared with BD (non-NSSI) group: *P < 0.05

## Discussion

This study identified the metabolic pathways associated with BD (NSSI) and assessed the important diagnostic and predictive indices of NSSI in BD. To the best of our knowledge, this is the first study to identify the differential metabolites of BD (NSSI) and BD (non-NSSI).

NSSI is a characteristic manifestation of BD, due to its rapidly increasing prevalence, NSSI requires more clinical attention [28]. In a previous study, 5.1% to 24% of people who inflict self-injury reported that they had initiated this behavior before age 11–13 years [29]. In this study, we observed that the onset age of patients in the BD (NSSI) group was lower than that of those in the BD (non-NSSI) group, which is consistent with previous results. Cutting, scratching, burning, hitting, and biting are some of the most commonly used methods of inflicting NSSI. Most self-injurers cut themselves using a sharp object, such as a knife or blade, mainly on the forearms, legs, and/or abdomen [30], which was consistent with our research. Studies confirmed that life stressors and adverse interpersonal experiences are associated with an increased risk of NSSI [31, 32], which is consistent with the present result. In the present study, we also found that sleep problems were most common in the BD (NSSI) group than in the BD (non-NSSI) group or healthy group. Previous research suggests that multiple sleep variables, including poor sleep quality and frequent nightmares, are associated with and are independent risk factors for NSSI [33, 34]. Therefore, we suspect that interventions that improve sleep quality and sleep duration or reduce life stress may concomitantly decrease the risk of NSSI.

A series of studies of BD demonstrated abnormalities of energy metabolism in patients with BD [35]**.** Several studies have confirmed that lipid metabolic disorders or abnormalities is concerned with neuropsychiatric disorders, such as BD, schizophrenia, and major depressive disorder. Previous studies confirm that there is a high prevalence of elevated triglycerides, cholesterol, low-density lipoprotein, and glucose levels and low high-density lipoprotein (HDL) level in patients with BD [36]. In this study, we found that HDL is a common differential metabolite in the BD (NSSI) and BD (non-NSSI) groups, which suggested the importance of lipid metabolism in BD, that is consistent with previous studies [36, 37]. Results showed that sphingolipids and glycerolipids were increased, whereas glycerophospholipids were decreased, in serum samples from patients with BD. Moreover, studies also showed that elevated lipid level is associated with smaller brain structures in patients with BD [38]. Future research is needed to verify the changes in the HDL levels of BD patients compared with those of healthy individuals and the specific mechanisms of lipid changes in the pathogenesis of BD.

The results of the present study indicate that 3-hydroxybutyric acid, pyruvic acid, oxidized glutathione, glyceryl, citrulline, creatinine, and β-glucose are characteristic markers of bipolar NSSI. Patients in the BD (NSSI) group showed higher lipid, 3-hydroxybutyric acid, pyruvic acid, citrulline, and creatinine levels and lower oxidized glutathione, glyceryl, and β-glucose levels than the healthy controls. Kamonwad et al. reported that patients with BD have increased salivary levels of glutathione and oxidized glutathione compared to controls [39]. Rosa et al. [40] documented decreased levels of glutathione and increased levels of glutathione disulfide in the plasma of patients with BD. Previous studies have also revealed higher serum levels of pyruvate and N-acetyl glutamate in patients with BD than in healthy controls [35, 41], which is consistent with the findings of this study. However, previous studies have insufficiently focused on correlation between the abovementioned metabolites and NSSI in patients with BD.

This study showed that the urea cycle and the glutamate metabolism pathway are significant metabolic pathways in BD (NSSI). For the urea cycle, also known as the ornithine cycle, when amino acids are metabolized in the body, ammonia is produced and subsequently synthesized into urea through. Studies have suggested that an abnormality in the urea cycle (or arginine metabolism) is associated with BD [42, 43]. The results of this study indicated that an abnormal urea cycle is associated with BD (NSSI). Xu et al. reported that state-related abnormalities in oxidative and glutamate metabolism are associated with BD [44, 45]. Increasing evidence suggests that changes in inflammatory mediators are involved in the pathogenesis of mood disorders [46, 47]. Meanwhile, studies have demonstrated links between alterations in inflammation and glutamate metabolism in mood disorders [48, 49]. This indicates that inflammatory mediators, glutamate metabolism and oxidative stress are closely related to the pathogenesis of BD(NSSI). Thus, therapeutic strategies targeting amino acid metabolism such as glutamate may be effective in patients with BD (NSSI), and increased inflammation as reflected in C-reactive protein levels may be helpful in the diagnosis of BD (NSSI).

In clinical practice, identifying specific diagnostic markers of NSSI in patients with BD will provide a strong basis for the recognition and treatment of NSSI. Thus, we also compared the metabolic differences between BD (NSSI) and BD (non-NSSI) groups. Five endogenous differential metabolites, including xanthine, niacinamide, adenosine, hypoxanthine, and histidine, were significantly higher in the BD (NSSI) group than in the BD (non-NSSI) group. Adenosine, a purine nucleoside, may contribute to the pathophysiology of mental disease by interacting with dopamine and glutamate receptors through A1 and A2A receptors; thus, modulating dopaminergic and glutamatergic neurotransmission [50, 51]. Zhang et al. reported that levels of the purines guanine and guanosine are decreased in the brains of patients with BD [52]. However, assessments of changes in purine and adenosine metabolism levels are lacking. This study shows that the important metabolic pathways associated with BD (NSSI) are the purine and methylhistidine metabolism pathways. The purinergic system is a critical neurotransmitter system with uric acid (UA) as its end-product. Recent studies have shown that the patients with BD have the highest UA levels among healthy controls and those with other mental disorders [53, 54], which is involved in the occurrence and development of mental illnesses such as BD and MDD [55, 56]. The purinergic system is involved in the neurodevelopment and pathophysiological processes of psychotic disorders, such as the genesis, differentiation of neurocytes and inflammation of neuroglial cells [57, 58]. Growing evidence suggests that oxidative stress and the purine/adenosine system play key roles in the development and progression of mental diseases, such as BD [59, 60]. We suggest that NSSI in patients with BD is related to an increase in oxidative stress levels. Post-mortem and imaging studies showed an increasingly complex interaction between the mitochondrial, purinergic, and oxidative systems, which are associated with psychiatric disorders [61]. These results suggest that an increase in purinergic-UA metabolism and oxidative stress levels may be a significant mechanism underpinning BD (NSSI), which may be related to mitochondrial dysfunction.

It has been hypothesized that gout and BD may share similar pathophysiological mechanisms, such as purinergic dysfunction. Previous research has shown that patients with BD have an increased risk of gout [62, 63]. We observed increased purine synthesis in the BD (NSSI) group in this study, indicating that the incidence of gout was higher in patients with BD (NSSI). This suggests that purine-UA metabolism is a potential therapeutic target in the treatment of BD (NSSI). As xanthine and hypoxanthine levels are elevated in BD (NSSI), allopurinol, an inhibitor of xanthine oxidase, is used to treat and prevent gout. Allopurinol and febuxostat [64], two potent inhibitors of UA accumulation, have demonstrated antimanic and antidepressant effects in clinical and preclinical studies, and may be used as add-on therapy for BD (NSSI) to reduce rates of self-injury. Hirota et al. suggested that adenosine modulator adjuvant therapy is more beneficial than a placebo in treating manic episodes of BD [65]. As adenosine levels were altered in this study, we suspect that adenosine modulator adjuvant therapy may be effective for BD (NSSI). These drugs can be used as potential therapeutic options for patients with BD (NSSI).

In this study, purine and amino acid metabolism were found to be altered in patients with BD (NSSI) compared to that in healthy controls, which is consistent with the findings of a previous study [66]. As the final metabolite of purine, UA acts on neurons presynaptically and postsynaptically, and on specific receptors in the glial cell membrane that can affect activities of other neurotransmitters involved in the pathophysiological process of mood disorders, including dopamine, GABA, glutamate, and serotonin [67]. It has been suggested that purinergic-UA metabolism is associated with glutamate metabolism, further affecting oxidative stress and is involved in the pathogenesis of NSSI, all of which are related to mitochondrial dysfunction [68–70]. This provides important evidence for the diagnosis and treatment of BD (NSSI). These findings provide a basis for further research on the pathogenesis of NSSI, and are highly significant with regard to the diagnosis, recognition, and treatment of NSSI.

## Limitations

This study has some limitations. First, the previous use of antidepressants and antipsychotic drugs may be a confounding factor for those eight participants with medication history. We did not control for the effects of psychotropic medications, which may affect plasma metabolite profiling. Second, the sample size was limited. Adequately powered studies are warranted in the future to confirm our preliminary conclusions. Therefore, further studies using a larger sample size of medication-free patients are required. Third, as the difference in metabolomics between the NSSI and non-NSSI groups can be attributed to various factors, such as the relationship between depressive symptom severity and outcome, we need to further evaluate this aspect in subsequent studies.

## Conclusion

This study demonstrated that purine and amino acid metabolism are greatly enhanced in BD with NSSI than that in BD without NSSI, providing evidence of the relationship between the purinergic system, glutamate metabolism, and the pathogenesis of NSSI in patients with BD. In addition, the results of this study indicate that xanthine, hypoxanthine, and adenosine may be potential biomarkers of NSSI in patients with BD. These findings suggest that abnormalities in the purinergic system, urea cycle, and glutamate metabolism play important roles in the pathogenesis of BD. Further investigations are needed to elucidate the relationship between purinergic-UA metabolism, amino acid cycling, and oxidative stress. In addition, future studies which are stringently designed with larger samples are required to validate our results and confirm our conclusions.

## Data Availability

All data used during the study appear in the submitted article.

## Abbreviations

BD: Bipolar disorder
NSSI: Non-suicidal self-injury
UA: Uric acid
HAMD: Hamilton Depression Scale
^1^H NMR: Proton nuclear magnetic resonance
PCA: Principal Component Analysis
iPCA: Interval principal component analysis
PLS-DA: Partial least-squares discriminant analysis
OPLS-DA: Orthogonal projections to latent structures discriminant analysis
OSC: Orthogonal signal correction
HDL: High-density lipoprotein
HC: Healthy control
